# Reconstructing propagation networks with temporal similarity

**DOI:** 10.1038/srep11404

**Published:** 2015-06-18

**Authors:** Hao Liao, An Zeng

**Affiliations:** 1 Guangdong Province Key Laboratory of Popular High Performance Computers, College of Computer Science and Software Engineering, Shenzhen University, Shenzhen 518060, P. R. China; 2 School of Systems Science, Beijing Normal University, Beijing 100875, P. R. China; 3 Institute of Information Economy, Alibaba Business School, Hangzhou Normal University, Hangzhou 310036, P. R. China; 4 Department of Physics, University of Fribourg, Chemin du Musée 3, CH-1700 Fribourg, Switzerland

## Abstract

Node similarity significantly contributes to the growth of real networks. In this paper, based on the observed epidemic spreading results we apply the node similarity metrics to reconstruct the underlying networks hosting the propagation. We find that the reconstruction accuracy of the similarity metrics is strongly influenced by the infection rate of the spreading process. Moreover, there is a range of infection rate in which the reconstruction accuracy of some similarity metrics drops nearly to zero. To improve the similarity-based reconstruction method, we propose a temporal similarity metric which takes into account the time information of the spreading. The reconstruction results are remarkably improved with the new method.

One of the key features in complex networks is the similarity between nodes^1^. The intrinsic similarity between nodes is one of the mechanisms driving the growth of networks^2^. Consequently, nodes in a network may appear to have some level of similarity in topology. An accurate estimation of nodes’ topology similarity is fundamental to many applications in network science, including link prediction^3^, personalized recommendation^4^, spurious link identification^5^,^6^, backbone extraction^7^,^8^,^9^, community detection^10^,^11^ and network coarse-graining^12^,^13^. However, how to estimate the topology similarity between nodes still remains a challenge in which the optimal solution depends significantly on the problems we are facing. For example, in recommender systems it has already been pointed out that a more effective similarity metric should be biased to small degree nodes to enhance diversity of the recommendation^4^. For the problem of spurious link identification^5^, the similarity metric should be combined with the betweenness index to avoid removing the important links connecting communities^14^. The concept of similarity is applied to compare sampled networks in order to detect damage in the original networks^15^.

The spreading, as an important dynamics in networks, has been applied to simulate many real processes including epidemic contagion^16^,^17^,^18^, cascading failure^19^, rumor propagation^20^,^21^,^22^, and others^23^,^24^,^25^. Recently, one fundamental problem about the spreading process attracts increasing attention: reconstructing propagation networks from observed spreading results^26^. In some real systems, partial data of the spreading process are visually available, but the underlying structure of the propagation network is not accessible. For example, the propagation of risk in financial systems^27^ and the diffusion of chemicals in neural systems^28^ are important dynamics processes for these systems. However, the inter-bank lending relations are commercial secrets^29^ and the synaptic connections between neurons are very difficult to detect^30^. Therefore, how to reconstruct the propagation network from the collected spreading data is very meaningful for understanding these real systems. Moreover, knowing the propagation networks can help us to hinder the propagation in the context of epidemic spreading. For instance, one effective way is to immunize the nodes that connecting different clusters in the propagation networks^31^.

Very recently, the compressed sensing theory has been introduced to infer the propagation networks^32^. This technique, though effective, has relatively high computational complexity which prevents its application in large scale networks. Real networks, especially in online social systems, can contain millions of nodes. An efficient algorithm should be based only on local information. To solve this problem, some local similarity metrics have been applied to inferring the propagation networks^33^. The basic idea is that the nodes’ similarity in the “infection pattern” is connected with their similarity in topology. In other words, nodes receiving similar information/virus in spreading are more likely to be connected in the propagation networks. However, the similarity-based methods only use the final spreading results as input information. In reality, one may be able to access more detailed spreading information even including the time stamp that records when the information/virus reached the node. If such information is used properly, it may significantly improve the inference accuracy.

Even though there are many problems, such as link prediction^3^ and personalized recommendation^4^, related to the network reconstruction, they are essentially different. In link prediction and personalized recommendation, the main task is to estimate the likelihood of a nonexisting link to be an existing link in the future^3^. A method that putting many future existing links on the top of the likelihood ranking has high accuracy. In network reconstruction, the accuracy is not the only focus. A well-performing method should also avoid high ranking of the false links that may result in significant difference between the reconstructed network and the real network. Therefore, one may reach completely different conclusions even if the same similarity method is applied to these two different types of problems^14^. In this context, the performance of the existing similarity metrics has to be reexamined when applied to network reconstruction.

In this paper, we first systematically study the performance of different similarity metrics in reconstructing the propagation networks. Some methods with high accuracy in predicting missing links perform very badly in reconstructing the propagation networks under some infection rates. We find that this is because these similarity metrics overwhelmingly suppress high degree nodes, so that the links are mostly connected to the nodes that are supposed to have low degree. Moreover, we find a phenomenon called “more is less”: when the infection rate is higher than the critical value, each information/virus covers a large part of the network, making the similarity metric fails to capture the local structure of the network. In order to solve this problem, we propose a temporal similarity metric to incorporate the time information of the spreading results. The simulation results in both artificial and real networks show that the reconstruction accuracy is remarkably improved with the new method.

## Results

### Problem Statement

We make use of the well-known Susceptible-Infected-Remove (SIR) model to simulate the spreading process on networks^34^. Although it is an epidemic spreading model, it has also been applied to model the information propagation process^35^. While we use here the terminology of news propagation, our results remain applicable to the epidemic spreading case.

A social network with *N* nodes and *E* links can be represented by an adjacency matrix *A*, with *A*_*ij*_ = 1 if there is a link between node *i* and *j*, and *A*_*ij*_ = 0 otherwise. In our model, each node has a probability *f* submitting a piece of news to the network. As there are *N* nodes in the network, finally there will be *f* × *N* pieces of news propagating in the network. The propagation of the news follows the rule of the SIR model: After news *α* is submitted (or received) by a node, it will infect each of this node’s susceptible neighbors with probability *μ*. After infecting its neighbors, the node is marked as recovered. During the spreading, we record all the news that each node receives and the time step when it happens. At the end, the information of news received by nodes is stored in a matrix *R*, with *R*_*iα*_ = 1 if *i* have received news *α*, and *R*_*iα*_ = 0 otherwise. When *R*_*iα*_ = 1, the time step at which *i* received *α* is recorded in *T*_*iα*_. In the simulation, we use parallel update of nodes’ status in the spreading. It means that the time step is updated after all infected nodes finish the attempt to infect neighbors. In next time step, all the newly infected nodes from last time step will attempt to infect their neighboring nodes. The main task is to use the information stored in *R* and *T* to rebuild the network *A*. The notations of important variables are presented in Table 1.

### Similarity metrics

The methods we used to reconstruct the network will be based on node similarity. The basic idea is that the nodes receiving many common news are similar and tend to link together in the network. Therefore, the similarity *s*_*ij*_ between node pair *ij* can be used to estimate the likelihood *L*_*ij*_ for two nodes to have a link in the network. With *R*, many similarity methods can be used to calculate the similarity between nodes. The performance of these methods have been extensively investigated in^36^. Here, we mainly consider four representative methods: Common Neighbors (CN)^1^, Jaccard (Jac)^37^, Resource Allocation (RA)^38^ and Leicht-Holme-Newman (LHN)^39^ Indices.

We select these four indices because we want to explore different type of similarity definitions. The CN and RA similarities are in favor of the high degree nodes. The Jaccard similarity reduces the advantage of high degree nodes by normalizing the number of common news with the size of the union of the received news. The LHN similarity punishes the high degree nodes even more than the Jaccard. By comparing the results of CN, Jac and LHN, we can investigate the influence of different penalty schemes (i.e. CN: no penalty; Jac: median penalty; LHN: strong penalty) on node degree on the network reconstruction results.

As we are able to get access to the information of the time step *T*_*iα*_ at which the news *α* is received by the node *i*, we can further improve the similarity with *T*_*iα*_. If two nodes receive the news at a closer time step, they are more likely to be connected in the network. Therefore, for each similarity method, we will design an improved method based on the temporal information of the news propagation. The improved methods are respectively Temporal Common Neighbors (TCN), Temporal Jaccard (TJac), Temporal Resource Allocation (TRA) and Temporal Leicht-Holme-Newman (TLHN) Indices. The detailed description of the methods can be seen in the Methods section.

### Metrics

We adopt three metrics to evaluate the performance of different methods. The first one is the standard metric of the area under the receiver operating characteristic curve (AUC)^40^. Each method above gives a score to all node pairs in the network, and the AUC represents the probability that a true link has a higher score than a nonexisting link. To obtain the value of the AUC, we pick a true link and a nonexisting link in the network and compare their scores. We randomly pick up *n* pairs of such links in total. The number of times that the real link has a higher similarity score *s*_*ij*_ than the nonexisting link is denoted as *n*_1_. Moreover, we use *n*_2_ to denote the number of times that the real link and the nonexisting link have the same score *s*_*ij*_. And the AUC value is then calculated as follows:


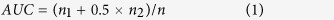


If links were ranked at random, the AUC value would be equal to 0.5. We tested different *n* value and find that *AUC* in different realization is already very stable after *n* > 10^4^. Therefore, we set *n* = 10^5^ in this paper.

The second and third metrics require the reconstruction of the network. The node pairs are ranked in descending order according to *s*_*ij*_, and *E* (we assume that we know roughly the number of real links in the network) top-ranked links are used to reconstruct the network. The precision of the reconstruction, as the second metric, can be assessed by the overlap of the links in the reconstructed network and the real network. If *m* out of *E* top-scoring links occur also in the real network of size *E*, precision is *m*/*E*. The precision metric can be regarded as a complementary measurement to AUC. The third metric is the Pearson correlation between node degree in the reconstructed network and the real network. In fact, AUC and precision measure the reconstruct performance computing on individual level, i.e. whether the top-ranked link exist or not in the network. The degree correlation, on the other hand, evaluates the methods in rather collective level, i.e. whether the methods can correctly infer the degree of nodes.

### Artificial networks

**Figure 1:**
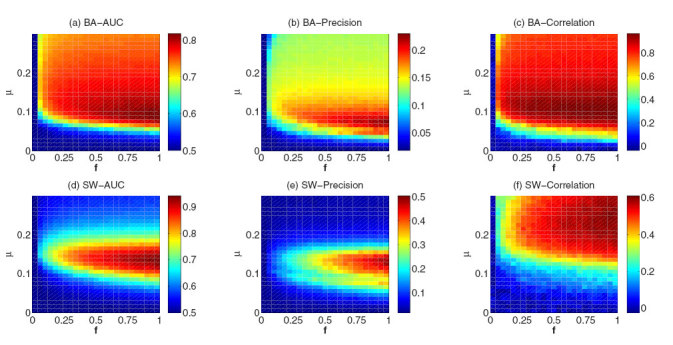
The *AUC*, *Precision* and *Degree correlation* in the parameter space ( *μ*, *f* ) for (**a**,**b**,**c**) BA networks (*N* = 500, 〈*k*〉 = 10) and (**d**,**e**,**f**) SW networks (*N* = 500, *p* = 0.1, 〈*k*〉 = 10) by using *CN* method. The results are averaged over 50 independent realizations.

We first analyze the methods in two classic artificial networks: (i) Small-World networks (SW), generated by the Watts-Strogatz model^41^ and (ii) Scale-free networks, generated by the Barabasi-Albert model (BA)^42^. The spreading process has two parameters: infection rate *μ* and news submission probability *f*. With the Common Neighbor (CN) method as an example (see the results of other methods in Fig. S1, Fig. S2 and Fig. S3 in the Supplementary Information), we study the influence of these two parameters on the network reconstruction results in Fig. 1. The *AUC*, precision and degree correlation in the parameter space (*μ*, *f*  ) for both BA and SW networks are shown. One can see that *μ* significantly affects the results in each panel. In BA networks, the optimal *μ* results in the highest *AUC*, and precision and degree correlation are nearly the same (around 0.1). However, in SW networks the optimal *μ* for *AUC* and precision is different from the optimal *μ* for degree correlation. More specifically, to achieve the highest *AUC* and precision, *μ* in SW needs to be around 0.15. However, the best *μ* for degree correlation is around 0.25. In^33^, it has already been pointed out that the optimal *μ* for AUC is roughly equal to 1/〈*k*〉. Different from *μ*, the effect of *f* on the results is monotonous. All the three metrics increase remarkably as *f* increases. After *f* is higher than a threshold, these three metrics are affected only slightly by *f* (see Fig. S4 in the SI for the dependence of the three metrics on *f*  ).

**Figure 2:**
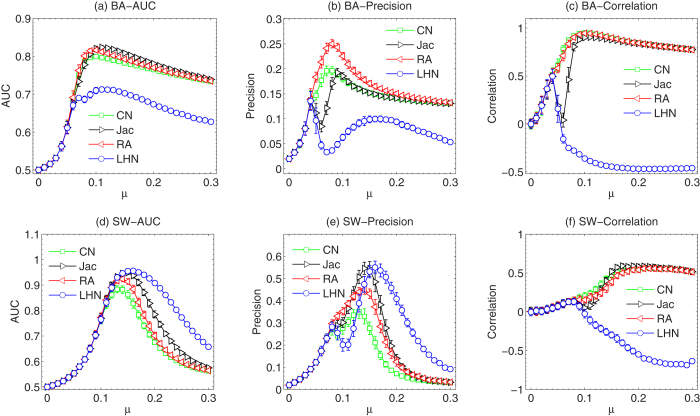
The dependence of the *AUC*, *Precision* and *Degree correlation* on *μ* with four different similarity methods in BA networks (*N* = 500, 〈*k*〉 = 10) and (**d**,**e**,**f**) SW networks (*N* = 500, *p* = 0.1, 〈*k*〉 = 10). We use *f* = 0.5 here. The results are averaged over 50 independent realizations.

We further compare the performance of different similarity methods. To this end, we present the dependence of *AUC*, precision and degree correlation on *μ* of CN, Jac, RA and LHN methods in Fig. 2. As in real systems, the observed propagation results are usually limited, we thus use a relatively small *f* in this figure, i.e. *f* = 0.5. As we discussed in Fig. 1, when CN is applied, one can observe a pronounced peak when tuning *μ*. The reason for this peak has already been explained in ref. 33. Here, the interesting phenomenon happens when different similarity methods are compared. For Jac and LHN, the peaks in AUC still exist. However, when precision and degree correlation are considered, the curves of these two metrics drop suddenly within a certain range of *μ* which we refer to as the “special range” of *μ*. The “special range” is actually due to two reasons: the similarity degeneracy and degree penalty of the similarity metrics. The similarity degeneracy mainly explains the “special range” in CN method. It means that there are some node pairs with the same similarity when *μ* is in the “special range” that one cannot set a simple threshold to cut top-*E* links to reconstruct the propagation network. In this case, many links need to be randomly selected from a large number of candidates, resulting in a low reconstruction precision. The degree penalty mainly explains the “special range” in Jac and LHN methods. These similarity metrics overwhelmingly suppress high degree nodes, so that the links are mostly connected to the nodes that are supposed to have low degree. A quantitative analysis and explanation of the “special range” are reported in SI.

**Figure 3:**
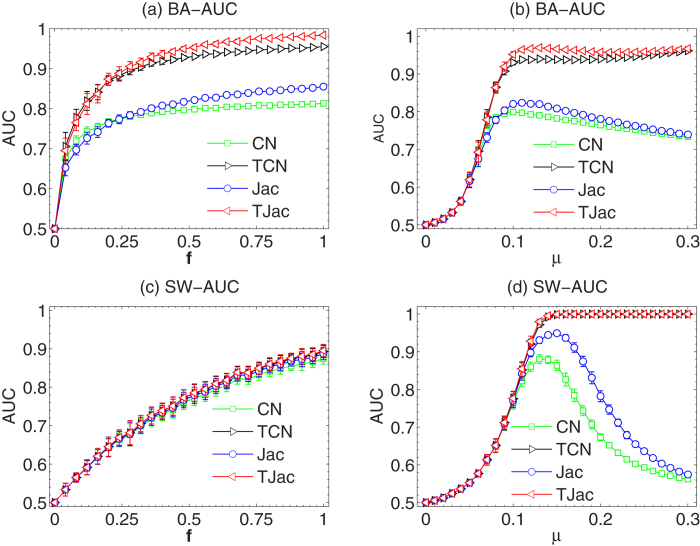
The dependence of the *AUC* on *f* with temporal similarity methods in BA and SW networks. We use *μ* = 1/〈*k*〉 in (**a**) and (**c**), and *f* = 0.5 in (**b**) and (**d**). The results are averaged over 50 independent realizations.

**Figure 4:**
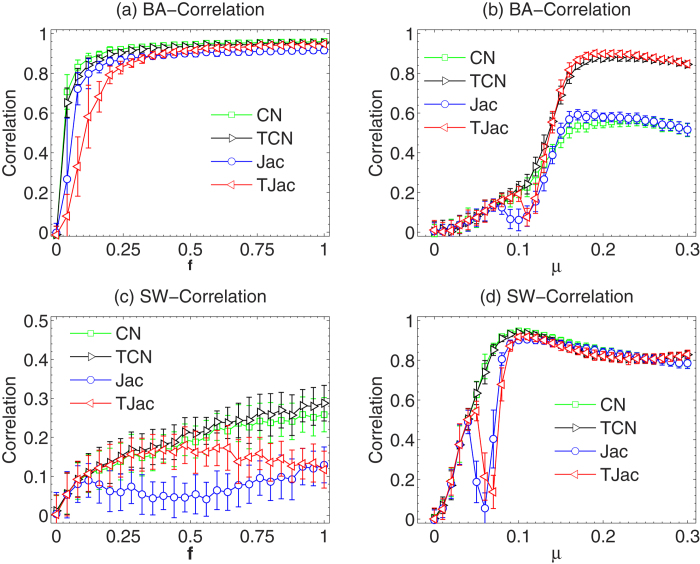
The dependence of *Degree correlation* on *f* with temporal similarity methods in BA and SW networks. We use *μ* = 1/〈*k*〉 in (**a**) and (**c**), and *f* = 0.5 in (**b**) and (**d**). The results are averaged over 50 independent realizations.

During the news propagation process, the time stamp when the news reaches each node is recorded. We thus used the temporal information of the news propagation to improve the existing similarity methods (see the Methods section). Here, we present the advantage of these temporal similarity methods in Fig. 3 and 4. In Fig. 3, we show the dependence of the AUC on *f* and *μ*. In Fig. 3(a)*μ* = 1/〈*k*〉 and one can see that TCN and TJac can significantly outperform CN and Jac, respectively (see the results of other temporal similarity methods in Fig. S5 in the SI). In Fig. 3(c)*μ* = 1/〈*k*〉 again, but the curves of the original similarity methods and the temporal similarity methods overlap, indicating the received news under this *μ* dominates the similarity. In Fig. 3(b, d), one interesting feature of the temporal similarity methods can be observed when tuning *μ*. When *μ* is large, the *AUC* of the classic similarity methods is very low. This is because the news proposed by every node can reach a large part of the networks, so that the news coverage can no longer reflect the topology information of the network. However, when TCN and TJac methods are applied, AUC can remain close to 1 even when *μ* is as large as 0.1. These results indicate that the temporal information is crucial to the network reconstruction from the propagation processes. However, we have to remark that, when *μ* is small, as we see in the Fig. 3, the temporal information cannot improve *AUC*.

In Fig. 4, we study the dependence of degree correlation on *f* and *μ* respectively when the temporal similarity methods are used. Clearly, the temporal similarity methods cannot improve the correlation and the special range of *μ* still exists. This is easy to understand as the degree correlation is mainly determined by the normalization factor of the similarity methods. Therefore, when selecting the temporal similarity method, one still needs to be very careful, as an inappropriate method may still result in a negative degree correlation and very low reconstruction accuracy.

As shown above, the different similarity metrics yield very different results when varying the spreading parameters. In practice, one needs to estimate the spreading parameter before selecting the most appropriate similarity metrics to reconstruct the network. For instance, in a social network context, *μ* can be estimated by the mean-field approximation of the epidemic spreading process. By fitting the evolution of the infected node number with the mean-field curve, one can roughly estimate the parameter *μ* in the mean-field model^43^,^44^. As for *f*, one can estimate it by *M*/(*N* * *t*) where *M* is the number of news proposed by users in *t* period of time, and *N* is the number of users in the social network. These three values are normally publicly accessible in real online systems.

### Real undirected networks

We further apply the methods to the real networks. Firstly, the methods are applied to real undirected networks. We consider nine empirical networks including both social networks and nonsocial networks: (i) Dolphin: an undirected social network of frequent associations between 62 dolphins in a community living off Doubtful Sound, New Zealand^45^. (ii) Word: adjacency network of common adjectives and nouns in the novel David Copperfield written by Charles Dickens^46^. (iii) Jazz: a music collaboration network obtained from the Red Hot Jazz Archive digital database. It includes 198 bands that performed between 1912 and 1940, with most of the bands from 1920 to 1940^47^. (iv) E.coli: the metabolic network of E.coli^48^. (v) USAir: the US air transportation network which publicly available dataset at http://vlado.fmf.uni-lj.si/pub/networks/data/default.htm. (vi) Netsci: a coauthorship network between scientists who published on the topic of network science^46^. (vii) Email: an email communication network^49^. (viii) TAP: a yeast protein binding network generated by tandem affinity purification experiments^50^. (ix) PPI: a protein-protein interaction network^51^. We only take into account the giant component of these networks. This is because a pair of nodes located in two disconnected components, their similarity scores will be zero according to CN and its variants.

The results of the similarity methods on these networks are reported in Table 2 in detail. Consistent with the results in the artificial networks, the temporal similarity methods significantly outperform the classic similarity methods (not necessarily in degree correlation). In Table 2, TJac outperforms TCN in both AUC and Precision. The results of TLHN and TRA methods are reported in Table S2. The special range is also observed when LHN methods is applied to real networks. For example, in the email network, the degree correlation drops to negative when *μ* > 0.1, and the precision value is significantly lowered (from 0.2 to 0.02). However, we also observe that Jac no longer leads to the sudden drop of correlation and precision in the real networks we considered. Comparing all the methods, the TRA method generally enjoys the highest accuracy.

### Real directed networks

The methods are also applied to real directed networks. We considered several real directed networks to validate our methods. Results of TCN and TJac are shown in Table 3 and results of TLHN and TRA methods are shown in Table S4. The networks include Prisoners (friendship network between prisoners, available dataset at http://www.casos.cs.cmu.edu/index.php), St. Marks FW (food web in St. Mark area collected by http://www.cosinproject.org/), C. elegans neural (neural network of C. elegans)^52^, C. elegans metabolic (metabolic network of C. elegans)^52^, and PB (hyper link between the blogs of politicians, available at http://incsub.org/ blogtalk/images/robertackland.pdf).

Like the undirected networks, the temporal similarity methods have a much higher AUC and precision than the classic similarity methods. However, one can also see that AUC and Precision in directed networks are on average lower than the undirected networks. This indicates that it is generally more difficult to reconstruct directed networks via similarity metrics. We also studied the effect of *μ* on the results in directed networks. We observe that the improvement of the temporal similarity methods becomes more significant when *μ* is larger. Moreover, the special zone of both the Jac and LHN methods exists when adjusting *μ* in directed networks. Taking the Neural network as an example, when LHN is applied and *μ* > 0.08, the degree correlation drops to negative and the precision decreases from 0.15 to 0.07. We remark that the results on other networks are similar.

We select real networks from diverse backgrounds in order to study the performance of the similarity methods in different situations. Table 2 and 3 show that the method with the highest accuracy is almost unchanged in different networks. This means that the performance of similarity methods with respect to the accuracy is robust. However, when the degree correlation is measured, the results depend more on the networks, as shown in Table 2 and 3. The degree correlation measures whether the node degree in the reconstructed network is correlated with the node degree in the real network. In this case, a method that performs well in one type of networks is not guaranteed to perform well in other types of networks. For example, if the degree distribution of the real network is very heterogeneous, CN would work better in recovering the node degree (as the nodes’ CN similarity score is proportional to their degree). If the degree distribution is homogeneous, Jac or LHN similarity measures may outperform CN in degree correlation due to the higher accuracy.

### Other similarity metrics

Besides the four similarity metrics, we tested some other similarity metrics such as the Cosine index (Cos)^53^, Hub depressed index (HDI)^38^, Hub promoted index (HPI)^42^, Sorensen index (SSI)^54^, Preferential attachment index (PA)^42^, Asymmetric Index (AS)^33^. For each method, we also study its temporal version. The description of these methods and their results are presented in SI (see Fig. S10 and Table S5, S6, S7).

We study the influence of different parameters (i.e. *N*, 〈*k*〉, *μ*) on the performance of different similarity metrics in network reconstruction. We find that the temporal similarity metrics can significantly outperform the corresponding traditional similarity metrics especially when *μ* is large. When 〈*k*〉 increases, the precision of both traditional similarity metrics and temporal similarity metrics tend to increase. When *N* increases, the precision of both traditional similarity metrics and temporal similarity metrics tend to decrease. However, when 〈*k*〉 and *N* increase, the temporal metrics constantly outperform the traditional metrics. Therefore, it is better to use the temporal similarity metrics to reconstruct networks.

When different similarity metrics are compared, we find that CN and RA indices have smaller drop of precision in the “special range” than the other similarity metrics such as LHN, SSI, HPI, HDI, Cos and Jac. This is because the latter group of metrics all has some form of punishment based on node degree. In LHN, the drop of precision in the special range is most significant. The “special range” effect is much less obvious when the temporal similarity metrics are used. In LHN, however, an observable drop of precision in the “special range” still exists. This is because the degree punishment is most severe in LHN. We then compare the results of different metrics on SW and BA networks. In SW networks, all the temporal metrics can reach a very high precision (close to 1) when *μ* is large. However, TRA method reaches the highest value later (i.e. a larger *μ* is needed) than the other methods. In BA networks, the THPI reaches a highest precision.

In summary, if the time information of the spreading is unknown, it is better to use RA and CN to reconstruct the network as their precision is not affected much by the “special range” effect. If the time information of the spreading is available, it is better to use THPI to reconstruct the network as it works similar to other metrics in heterogeneous networks and it works best in homogeneous networks.

## Discussion

In this paper, we applied several standard similarity metrics to reconstruct the propagation network based on the observed spreading results. We find that even though some similarity methods such as Jaccard and LHN perform well in link prediction, they may cause problems when they are used to reconstruct networks, as they punish too much the nodes received many news and assign a large number of links to the nodes that supposed to have low degree. We find that the resource allocation method not only has high reconstruction accuracy, but also results in similar network structural properties as the original network. Finally, we take into account the temporal information of the propagation process, and we find that such information can significantly improve the reconstruction accuracy of the existing similarity methods, especially when the infection rate is large.

The value range of the infection rate in which the performance of Jaccard and LHN suddenly drops is denoted as a “special range” in this paper. The special range cannot be observed if one uses AUC to assess the network inference results. It can only be seen when one picks up the top ranking predicted links and uses them to reconstruct the network. This means that in the “special range”, even though existing links are still highly ranked in general by these link prediction algorithms (high AUC), only few links are actually located in the top-ranking (low precision). Therefore, the discovery of this “special range” not only gives warning information that a well-performed similarity method is not for sure effective in all difference cases, but also highlights the fact that precision of the predicted links needs to be measured when judging the performance of the similarity methods. This is also an important message for the link prediction research in which AUC is usually adopted as the only metric to evaluate the prediction results.

Some problems still remain unsolved. For example, our methods now require full time information. When only partial time information is available, the temporal similarity methods must be modified. In addition, our work only considers the simplest epidemic spreading model. Other more realistic models describing the disease contagion and information propagation need to be examined^55^. Furthermore, similar problems in other fields also need to be addressed. For instance, most link prediction methods are based on the observed network topology. When the time information of the observed links is available, the similarity methods should be modified accordingly to incorporate the temporal information of the network. Node similarity is also a basic network feature for community detection. Improving the community detection accuracy with the time information could be important problem. We believe that our work will inspire possible solutions to the above mentioned problems in the near future.

## Methods

The original similarity methods and the improved ones based on time information are listed below.

(i) *Common Neighbours (CN)* The common neighbor index is the simplest one to measure node similarity by directly counting the overlap of news received, namely


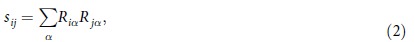


where *R*_*iα*_ = 1 if *i* have received news *α*, and *R*_*iα*_ = 0 otherwise.

(ii) *Temporal Common Neighbours (TCN)* This method, based on the common neighbor index, takes into account the time steps difference between two nodes receiving the news in common. The formula reads


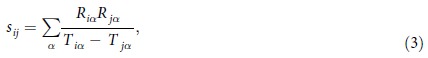


where *T*_*iα*_ records the time step at which *i* received *α*. If two nodes receive the news at a closer time step, they are more likely to be connected in the network.

(iii) *Jaccard Index (Jac)* This index was proposed by Jaccard^37^ over a hundred years ago. It can prevent the large degree nodes from having too high similarity with other nodes. The index is defined as


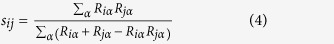


(iv) *Temporal Jaccrad Index (TJac)* The Jaccard index can also be improved by *T*_*iα*_ as


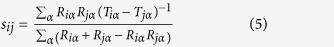


(v) *Resource Allocation Index (RA)* The similarity between *i* and *j* is defined as the amount of resource *j* received from *i*^38^, which is


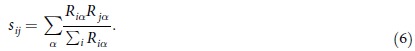


(vi) *Temporal Resource Allocation Index (TRA)* The improved RA method reads


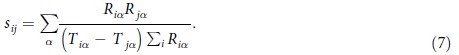


(vii) *Leicht-Holme-Newman Index (LHN)* This index assigns high similarity to node pairs that have many common neighbours compared to the expected number of such neighbours^39^. It is defined as


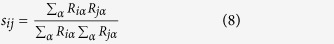


(viii) *Temporal Leicht-Holme-Newman Index (TLHN)* Similar to the above three improved methods, the formula is


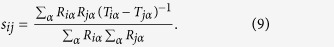


In all the temporal similarity methods above, we set (*T*_*iα*_ − *T*_*jα*_)^−1^ = 0 when *T*_*iα*_ = *T*_*jα*_. In this case, *i* is definitely not the node that passes the news to *j*, so *i* and *j* are unlikely to be connected in the networks. We pose this setting as it applies to our step-by-step spreading model. Note that in other problems such as link prediction and recommendation, the case of *T*_*iα*_ = *T*_*jα*_ may have to be treated differently.

## Additional Information

**How to cite this article**: Liao, H. and Zeng, A. Reconstructing propagation networks with temporal similarity. *Sci. Rep*. **5**, 11404; doi: 10.1038/srep11404 (2015).

## Supplementary Material

Supplementary Information

## Figures and Tables

**Table 1:** Variable notations in this paper.

Variable	Description
N	Number of nodes in the network
E	Number of links in the network
A	Adjacency matrix of the network
〈*k*〉	Average degree of the network
*f*	Probability of a node to submit a news in each step
*μ*	Infection rate of the spreading
*R*_*iα*_	*R*_*iα*_ = 1 means node *i* received news *α*, otherwise, *R*_*iα*_ = 0
*T*_*iα*_	The time step at which node *i* received news *α*
*s*_*ij*_	The similarity between node pair *ij*
*d*_*ij*_	The total number of news received by each node pair *ij*

**Table 2:** Basic properties of real undirected networks and the performance of the CN, TCN, Jac and TJac methods on these networks.

Network	Basic properties		AUC				Precision					Degree correlation			
	N	E	CN	TCN	Jac	TJac	*P*_0_	CN	TCN	Jac	TJac	CN	TCN	Jac	TJac
Dolphins	62	159	0.78	0.96	0.83	**0.97**	0.08	0.34	0.66	0.38	**0.74**	0.66	0.76	0.70	**0.84**
Word	112	425	0.80	0.92	0.80	**0.93**	0.07	0.30	0.54	0.30	**0.55**	0.76	0.81	0.76	**0.87**
Jazz	198	2742	0.79	0.86	0.79	**0.86**	0.14	0.41	0.52	0.42	**0.53**	**0.85**	0.82	0.85	0.82
E. coli	230	695	0.87	0.94	0.89	**0.97**	0.03	0.32	0.52	0.33	**0.53**	0.83	0.79	**0.83**	0.79
USAir	332	2126	0.91	0.93	0.91	**0.94**	0.04	**0.52**	0.50	0.51	0.50	0.82	**0.84**	0.82	0.84
Netsci	379	914	0.86	0.98	0.97	**0.99**	0.01	0.21	0.61	0.44	**0.84**	0.50	0.63	0.64	**0.88**
Email	1133	5451	0.83	0.92	0.83	**0.93**	0.01	0.11	0.39	0.11	**0.40**	0.78	0.85	0.78	**0.85**
TAP	1373	6833	0.82	0.93	0.89	**0.99**	0.01	0.18	0.55	0.26	**0.58**	0.69	0.76	0.75	**0.78**
PPI	2375	11693	0.89	0.94	0.92	**0.97**	0.00	0.29	0.34	0.29	**0.35**	**0.79**	0.75	0.79	0.75

The parameters are set as *μ* = 2/〈*k*〉 and *f* = 0.5. We select a relatively large *μ* because the performance difference between traditional similarity metric and temporal similarity metric becomes more significant under large *μ*, as shown in Fig. 4. The *P*_0_ is calculated with 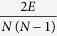, denoting the baseline precision value if the network is reconstructed at random. The similarity method with the best performance in each network is highlighted in bold font. The results are averaged over 50 independent realizations. The standard deviations are very small and presented in SI Table S1.

**Table 3:** Basic properties of real directed networks and the performance of the CN, TCN, Jac and TJac methods on these networks.

Networks	Basic properties		AUC				Precision					Degree correlation			
	N	E	CN	TCN	Jac	TJac	*P*_0_	CN	TCN	Jac	TJac	CN	TCN	Jac	TJac
Prisoners	67	182	0.72	0.81	0.80	**0.84**	0.04	0.21	0.47	0.41	**0.58**	0.57	0.69	0.68	**0.73**
SM-FW	54	356	0.65	**0.67**	0.63	0.66	0.12	0.25	**0.29**	0.24	0.28	**0.66**	0.64	0.33	0.33
Neural	297	2359	0.72	0.79	0.73	**0.81**	0.03	0.14	0.25	0.14	**0.29**	**0.68**	0.59	0.55	0.51
Metabolic	453	2040	0.68	0.70	0.70	**0.72**	0.01	0.09	0.14	0.14	**0.23**	0.54	0.64	0.60	**0.71**
PB	1222	19090	0.84	0.86	0.84	**0.86**	0.01	0.15	0.25	0.16	**0.25**	**0.81**	0.80	0.80	0.80

The parameters are set as *μ* = 2/〈*k*〉 and *f* = 0.5. We select a relatively large *μ* because the performance difference between traditional similarity metric and temporal similarity metric becomes more significant under large *μ*, as shown in Fig. 4. The *P*_0_ is calculated with 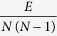, denoting the baseline precision value if the network is reconstructed at random. The similarity method with the best performance in each network is highlighted in bold font. The results are averaged over 50 independent realizations. The standard deviations are very small, and presented in SI Table S3.
