# Supplementary material for: Reconstructing propagation networks with temporal similarity
Source: Sci Rep. 2015 Jun 18;5:11404. doi: 10.1038/srep11404 (PMC4471885; doi:10.1038/srep11404)
Supplement: Supplementary Information [file srep11404-s1.pdf]

Supplementary Information
Reconstructing propagation networks with temporal similarity

Hao Liao and An Zeng

I. SI NOTES

A. "Special range".

The "special range" is actually due to two reasons: the similarity degeneracy and degree penalty of the similarity metrics. In order to understand why the "special range" exists when CN similarity is used, we take a detailed look at the similarity matrix under different infection rate μ. In our method, we need to take the E node pairs (E is the number of links in the true network) with the highest similarity and add links between each node pair to obtain the reconstructed network. When μ is in the special range, we find that there is a serious similarity degeneracy problem when we try to pick up the top E node pairs. It means that there are so many node pairs with the same similarity that one cannot use a simple similarity threshold to cut the similarity and obtain exact top E node pairs. We sort the similarity values in descending order and consider two adjacent similarity Sc_1 and Sc_2 such that $E_1 < E$ node pairs will be obtained when Sc_1 is used as the threshold and $E_2 > E$ node pairs will be obtained when Sc_2 is used as the threshold. In our method, Sc_1 is used in this case and $E - E_1$ node pairs are randomly selected from the node pairs with similarity Sc_2. We checked the value of $E_2 - E_1$, it is usually very large (e.g. larger than 10^4). Therefore, these $E - E_1$ node pairs needs to be randomly selected from a large number of candidates, resulting in a low reconstruction precision. To show this, we plot the dependence of $E - E_1$ on μ in Fig. S7. One can see that in SW, $E - E_1$ indeed suddenly increases in the "special range" . The increase of $E - E_1$ in BA is smaller, so we only observe a small drop of precision in the special range in this network in Fig. S7.

The similarity degeneracy in the "special range" is related to the spreading coverage under different μ. When μ is small, the spreading can only propagate very limited number of steps. Most of node pairs are with zero similarity. As μ increases, more nodes are with nonzero similarity and the similarity degeneracy becomes less serious. However, when μ is around the critical infection rate, the spreading starts to propagate locally, making many node pairs receive one or two common news. The similarity degeneracy becomes serious again. As μ further increases (larger than the "special range"), spreading propagates globally and the similarity between node pairs becomes well separated. This problem in Jac and LHN is less serious because they effectively reduce the similarity degeneracy by some normalization (but we can still observe an increase of $E - E_1$ in the "special range"). However, the normalization in these methods may cause strong penalty on large degree nodes, contributing also to the drop of precision in the "special range".

To show this, we denote the number of news the node i received as d_i and the degree of node i in the network as k_i. We first study the correlation between d_i and k_i under different infection rate μ. The result is shown in Fig. S8. In BA network, one can see that the correlation first increases with μ when μ is small. After reaching a highest value, the correlation starts to decrease. The "special range" happens when the correlation is very high. The behavior of the correlation between d_i and k_i is similar in SW. However, the "special range" happens when the correlation is around 0.25 (but not the highest).

Based on the results in Fig. S8, we conjecture the reason that forms the "special range" in Jac and LHN is as follows:

- **μ smaller than the "special range":** When μ is low, the spreading can only cover a small number of nodes. As the spreading information is so little (the similarity matrix is very sparse), it is natural that precision of the network reconstruction is low. As μ increases, the spreading covers more and more nodes and the similarity matrix becomes denser. The network reconstruction precision gradually increases with μ.

- **μ within the "special range":** As μ increases in BA networks, the correlation between d and k increases as well. The LHN metric computes the similarity between two nodes by the number of their common news over the product of their received news number (i.e. $d_i d_j$). When d becomes more strongly correlated with k, the large degree nodes will be punished and finally have very small number of links in the reconstructed network. This effect is absent when μ is small because the correlation d and k is very low in that case (e.g. lower than 0.25). The punishment from $d_i d_j$ is not concentrated on large degree nodes. In SW networks, as the correlation between d and k is not very high and there is no hub with extremely large degree in SW networks, the effect of the punishment is weaker and the drop of precision is less obvious in the "special range".

- **μ larger than the "special range":** when μ becomes even larger, the spreading can reach the small degree nodes more frequently. In this case, we can obtain meaningful similarity relation between the small degree nodes and other nodes. Therefore, the correctly predicted links of the small degree nodes will significantly increases with μ when μ is large enough. This could be the reason why the precision increases again after the "special range".

In order to prove the conjecture above, we carry out more simulation. We consider a group of small degree nodes consisting of the all nodes with the lowest degree in the network, and a group of large degree nodes consisting of all the nodes with the highest degree nodes. As degree distribution is homogeneous in SW, the small degree nodes group has similar number of nodes as the large degree node group. However, in BA, there is only one node with the highest degree. Therefore, we consider top-10 nodes with highest degree in BA. We study the relation between the number of correctly predicted links of different groups and the infection rate μ in Fig. S9.

Our conjecture above is proved by Fig. S9. In Fig. S9(d) (i.e. LHN is used in BA), the correctly predicted links (denoted as N_s) are mainly the links connecting to large degree nodes when μ is small. When μ is in the special range, the large degree nodes are punished and N_s drop to 0 due to the high correlation between k and d. Since some small degree nodes are connecting to these large degree nodes, their N_s are slightly punished as well. Therefore, we also observe a small drop of N_s for the small degree nodes. When μ is further increased, N_s for large degree nodes stay zero, but N_s for small degree nodes increases a lot since the spreading can reach the small degree nodes more frequently and result in a denser and more meaningful similarity matrix for prediction. The final drop of the number of correctly predicted links (when μ is very large) is because the spreading cover almost the whole network, the similarity extracted from the spreading can no longer reflects meaningful structural information of the network.

The results of LHN in SW are similar (see fig. S9(c)). As the degree is homogeneous in SW, the punishing effect is less obvious than BA. However, we can still observe that the large degree nodes start to be punished in the "special range" and small degree nodes start to have many correctly predicted links when μ is larger than "special range" . Since there is no penalty term in CN, there is no sudden drop of N_s of the large degree nodes in both Fig. S9(a)(d). The results of Jac are between CN and LHN, as shown in Fig. S9(b)(e).

B. Other similarity metrics

We study the influence of different parameters (i.e. N, $\langle k\rangle$, μ) on the performance of different similarity metrics in network reconstruction, as shown below in Fig. S10. We consider eight similarity metrics including the common neighbor index (CN), Jaccard index (Jac), Cosine index (Cos), Hub depressed index (HDI), Hub promoted index (HPI), Sorensen index (SSI), Resource allocation index (RA), and Leicht-Holme-Newman index (LHN). For each method, we also study its temporal version. The description of these methods is as follows.

(i) *Cosine Index (Cos)* It also named the Salton index [1], which is defined as

$$s_{ij} = \frac{\sum_\alpha R_{i\alpha}R_{j\alpha}}{\sqrt{\sum_\alpha R_{i\alpha}\sum_\alpha R_{j\alpha}}}. \tag{1}$$

(ii) *Temporal Cosine Index (TCos)* The Cos index can be improved by $T_{i\alpha}$ as

$$s_{ij} = \frac{\sum_\alpha R_{i\alpha}R_{j\alpha}(T_{i\alpha}-T_{j\alpha})^{-1}}{\sqrt{\sum_\alpha R_{i\alpha}\sum_\alpha R_{j\alpha}}}. \tag{2}$$

(iii) *Sorensen Index (SSI)* This index is used mainly for ecological community data [2], and is defined as

$$s_{ij} = \frac{2\times\sum_\alpha R_{i\alpha}R_{j\alpha}}{\sum_\alpha R_{i\alpha}+\sum_\alpha R_{j\alpha}}. \tag{3}$$

(iv) *Temporal Sorensen Index (TSSI)* The improved sorensen index is defined as

$$s_{ij} = \frac{\sum_\alpha R_{i\alpha}R_{j\alpha}(T_{i\alpha}-T_{j\alpha})^{-1}}{\sum_\alpha R_{i\alpha}+\sum_\alpha R_{j\alpha}}. \tag{4}$$

(v) *Hub Promoted Index (HPI)* This index is proposed for quantifying the topological overlap of pairs of substrates in metabolic networks [3], and is defined as

$$s_{ij} = \frac{\sum_\alpha R_{i\alpha}R_{j\alpha}}{min\{\sum_\alpha R_{i\alpha},\sum_\alpha R_{j\alpha}\}}. \tag{5}$$

(vi) *Temporal Hub Promoted Index (THPI)* This index is defined as

$$s_{ij} = \frac{\sum_\alpha R_{i\alpha} R_{j\alpha} (T_{i\alpha} - T_{j\alpha})^{-1}}{min\{\sum_\alpha R_{i\alpha}, \sum_\alpha R_{j\alpha}\}}. \tag{6}$$

(vii) *Hub Depressed Index (HDI)* There is a measure with the opposite effect on hubs [4]. It is defined as

$$s_{ij} = \frac{\sum_\alpha R_{i\alpha} R_{j\alpha}}{max\{\sum_\alpha R_{i\alpha}, \sum_\alpha R_{j\alpha}\}}. \tag{7}$$

(viii) *Temporal Hub Depressed Index (THDI)* It is defined as

$$s_{ij} = \frac{\sum_\alpha R_{i\alpha} R_{j\alpha} (T_{i\alpha} - T_{j\alpha})^{-1}}{max\{\sum_\alpha R_{i\alpha}, \sum_\alpha R_{j\alpha}\}}. \tag{8}$$

(ix) *Preferential Attachment (PA)* Based on the well-known Preferential Attachment process mechanism [3], the likelihood score for two nodes to have a link can be calculated as

$$s_{ij} = \sum_\alpha R_{i\alpha} \sum_\alpha R_{j\alpha}. \tag{9}$$

(x) *Temporal Preferential Attachment (TPA)* It can be expressed as

$$s_{ij} = \frac{\sum_\alpha R_{i\alpha} \sum_\alpha R_{j\alpha}}{T_{i\alpha} - T_{j\alpha}}. \tag{10}$$

(xi) *Asymmetric Index (AS)* This measure is for inferring the network topology in directed networks [5]. Mathematically, it can be expressed as

$$s_{ij} = \frac{\sum_\alpha R_{i\alpha} R_{j\alpha}}{\sum_\alpha R_{i\alpha}}. \tag{11}$$

(xii) *Temporal Asymmetric Index (TAS)* Similarly, it can be expressed as

$$s_{ij} = \frac{\sum_\alpha R_{i\alpha} R_{j\alpha} (T_{i\alpha} - T_{j\alpha})^{-1}}{\sum_\alpha R_{i\alpha}}. \tag{12}$$

We find that the temporal similarity metrics can significantly outperform the corresponding traditional similarity metrics especially when μ is large. This is consistent with the findings in the paper. When $\langle k \rangle$ increases, the precision of both traditional similarity metrics and temporal similarity metrics tend to increase. When N increases, the precision of both traditional similarity metrics and temporal similarity metrics tend to decrease. However, when $\langle k \rangle$ and N increase, the temporal metrics constantly outperform the traditional metrics. Therefore, it is better to use the temporal similarity metrics to reconstruct networks.

When different similarity metrics are compared, we find that CN and RA indices have smaller drop of precision in the "special range" than the other similarity metrics such as LHN, SSI, HPI, HDI, Cos and Jac. This is because the latter group of metrics all has some form of punishment based on node degree. In LHN, the drop of precision in the special range is most significant. The "special range" effect is much less obvious when the temporal similarity metrics are used. In LHN, however, an observable drop of precision in the "special range" still exists. This is because the degree punishment is most severe in LHN. We then compare the results of different metrics on SW and BA networks. In SW networks, all the temporal metrics can reach a very high precision (close to 1) when μ is large. However, TRA method reaches the highest value later (i.e. a larger μ is needed) than the other methods. In BA networks, the THPI reaches a highest precision.

In summary, if the time information of the spreading is unknown, it is better to use RA and CN to reconstruct the network as their precision is not affected much by the "special range" effect. If the time information of the spreading is available, it is better to use THPI to reconstruct the network as it works similar to other metrics in heterogeneous networks while works best in homogeneous networks.

II. SI FIGURES

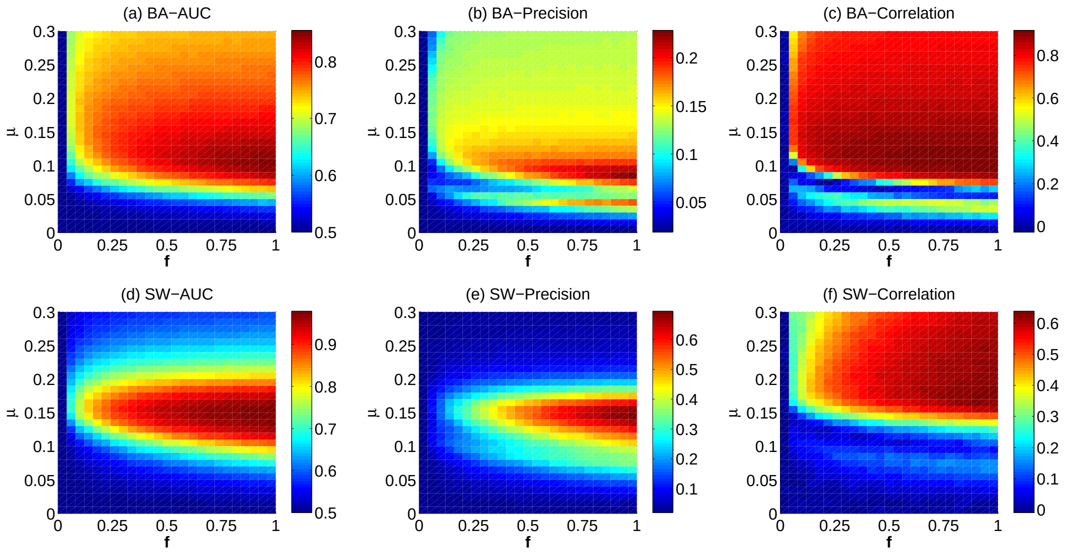

Fig. S1 The *AUC*, *Precision* and *Degree correlation* in the parameter space (μ, f) for (a,b,c) BA networks ($N = 500$, $\langle k \rangle = 10$) and (d,e,f) SW networks ($N = 500$, $p = 0.1$, $\langle k \rangle = 10$) by using *Jac* method. The results are averaged over 50 independent realizations.

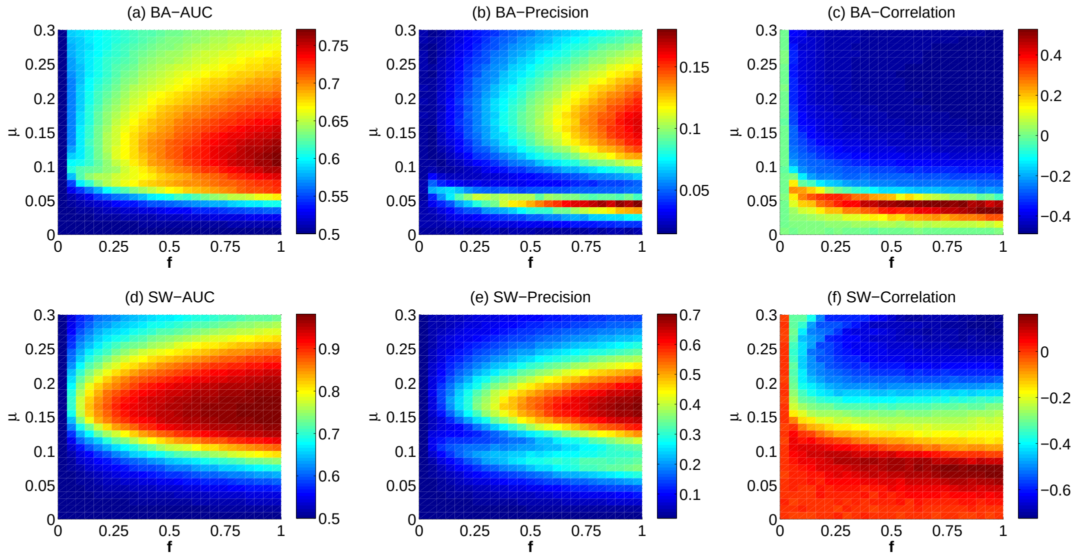

Fig. S2 The *AUC*, *Precision* and *Degree correlation* in the parameter space (μ, f) for (a,b,c) BA networks ($N = 500$, $\langle k \rangle = 10$) and (d,e,f) SW networks ($N = 500$, $p = 0.1$, $\langle k \rangle = 10$) by using *LHN* method. The results are averaged over 50 independent realizations.

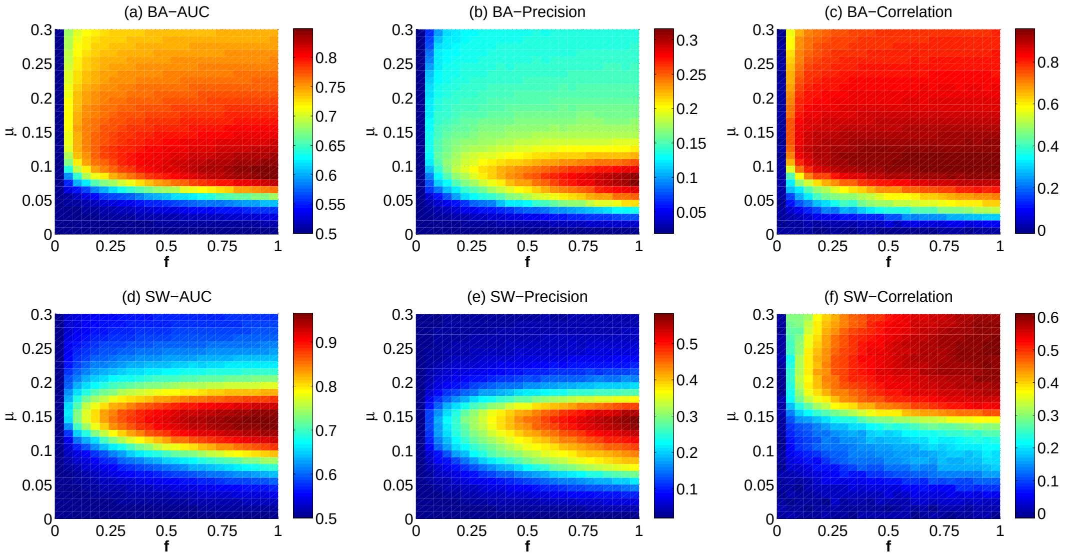

Fig. S3 The *AUC*, *Precision* and *Degree correlation* in the parameter space (μ, f) for (a,b,c) BA networks ($N = 500$, $\langle k \rangle = 10$) and (d,e,f) SW networks ($N = 500$, $p = 0.1$, $\langle k \rangle = 10$) by using *RA* method. The results are averaged over 50 independent realizations.

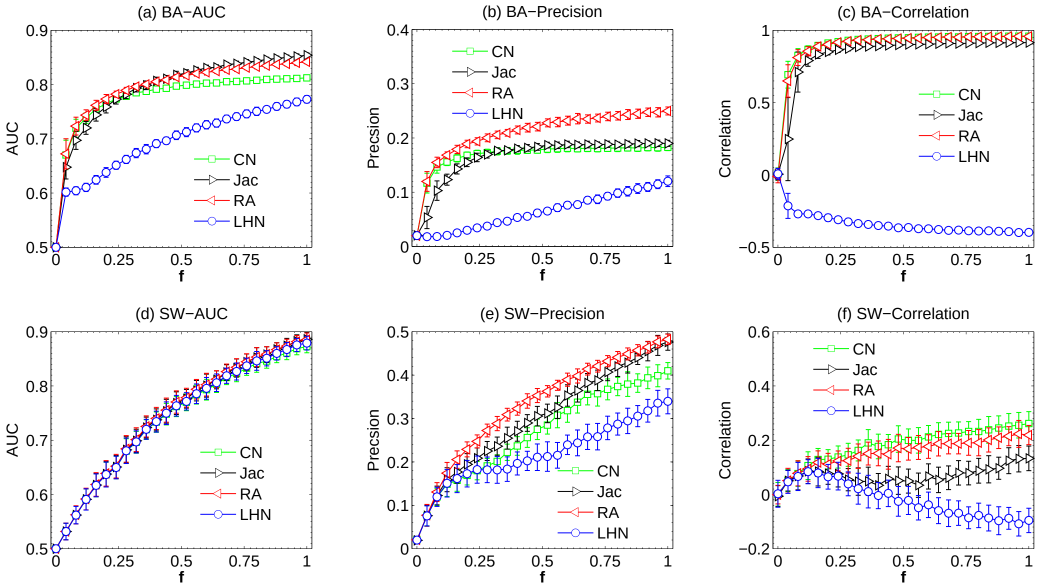

Fig. S4 The dependence of the *AUC*, *Precision* and *Degree correlation* on f with four different similarity methods for (a,b,c) BA networks ($N = 500$, $\langle k \rangle = 10$) and (d,e,f) SW networks ($N = 500$, $p = 0.1$, $\langle k \rangle = 10$). We use $\mu = 1/\langle k \rangle$ here. The results are averaged over 50 independent realizations.

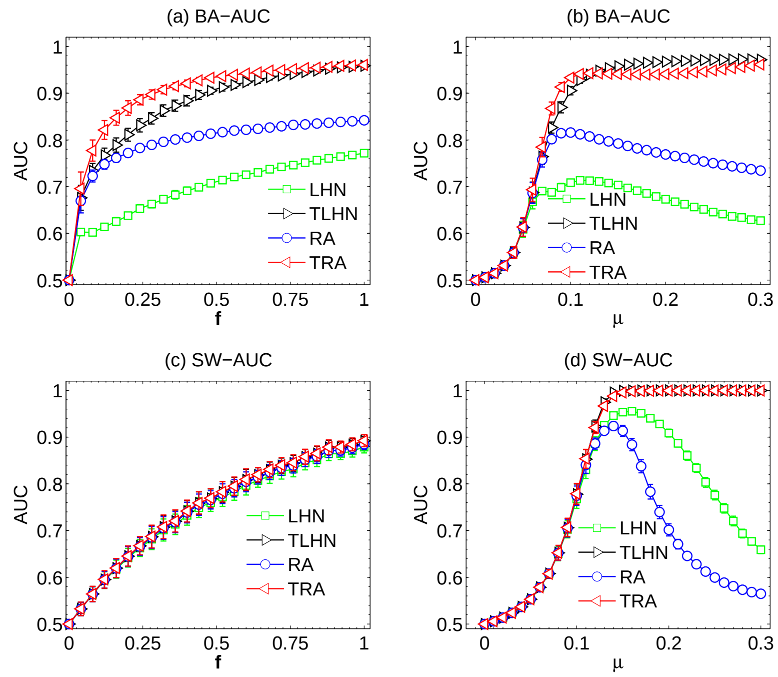

Fig. S5 The dependence of the *AUC* on f and μ separately with temporal-based similarity methods in BA and SW networks. We use $\mu = 1/\langle k \rangle$ in (a) and (c), and $f = 0.5$ in (b) and (d). The results are averaged over 50 independent realizations.

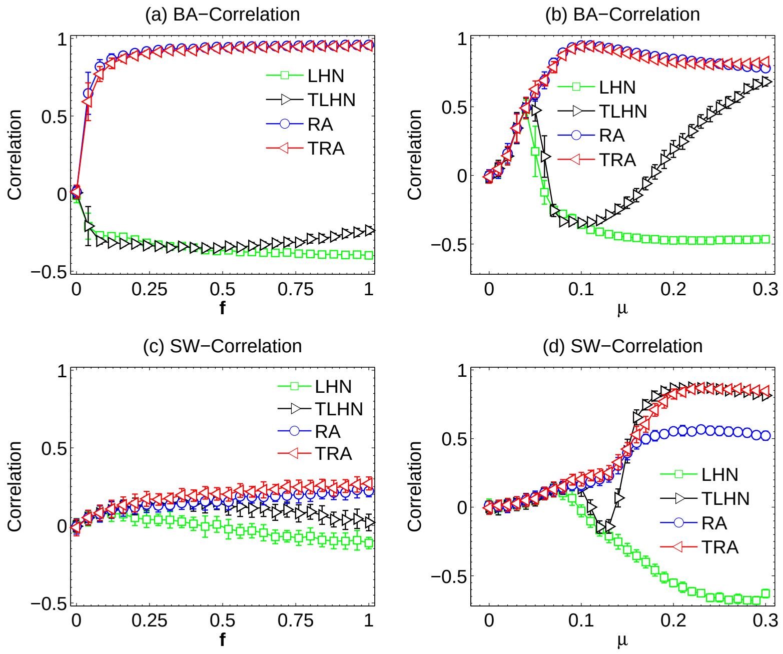

Fig. S6 The dependence of *Degree correlation* on f and μ separately with temporal-based similarity methods in BA and SW networks. We use $\mu = 1/\langle k \rangle$ in (a) and (c), and $f = 0.5$ in (b) and (d). The results are averaged over 50 independent realizations.

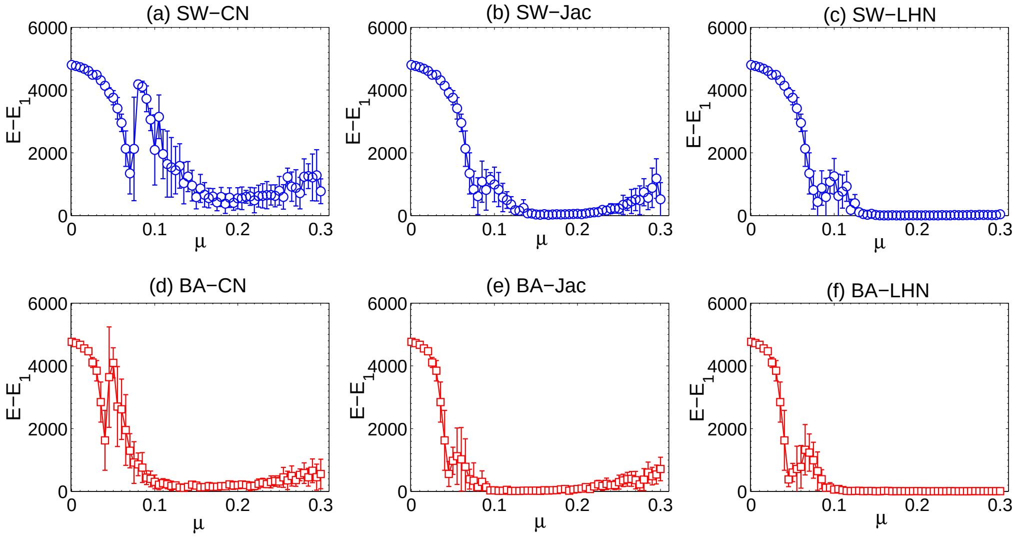

Fig. S7 The dependence of $E - E_1$ on μ when different similarity methods are used in SW and BA networks. The network parameters are the same as those used in Fig. 1 in the paper.

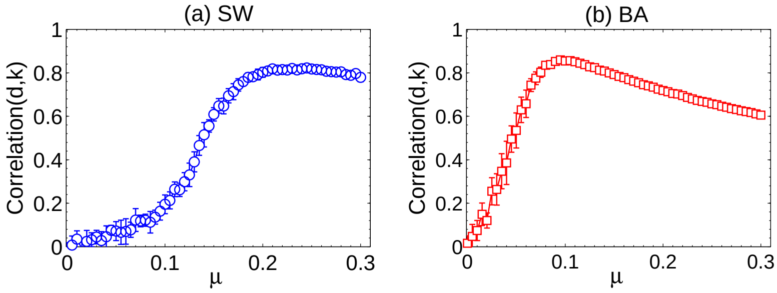

Fig. S8 The dependence of the correlation between d and k on μ (the blue curve), and the dependence of the precision on μ (the red curve). The similarity used for reconstructing the network is the LHN similarity. The network parameters are the same as the ones used in Fig. 1 in the paper.

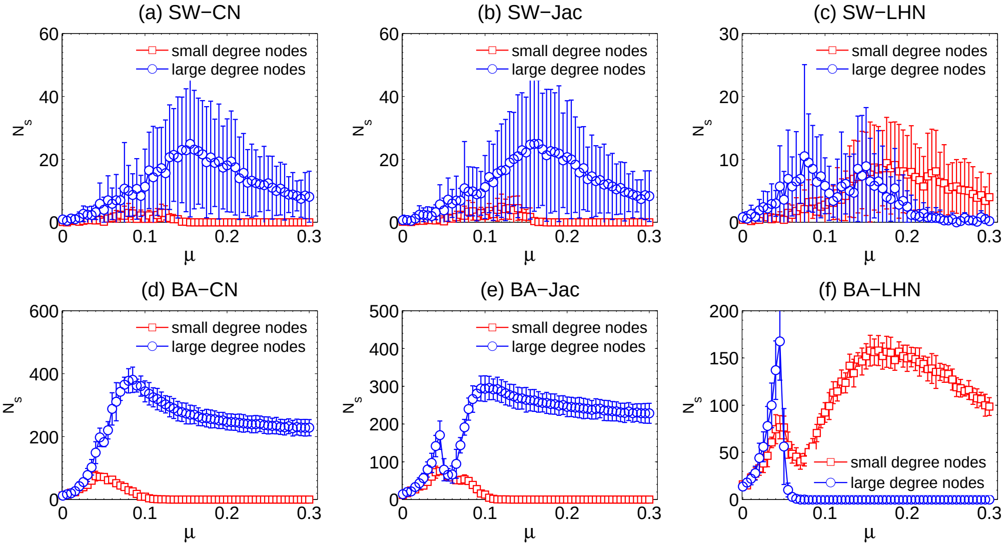

Fig. S9 The relation between the number of correctly predicted links (Ns) of different groups and the infection rate μ. The network parameters are the same as the ones used in Fig. 1 in the paper.

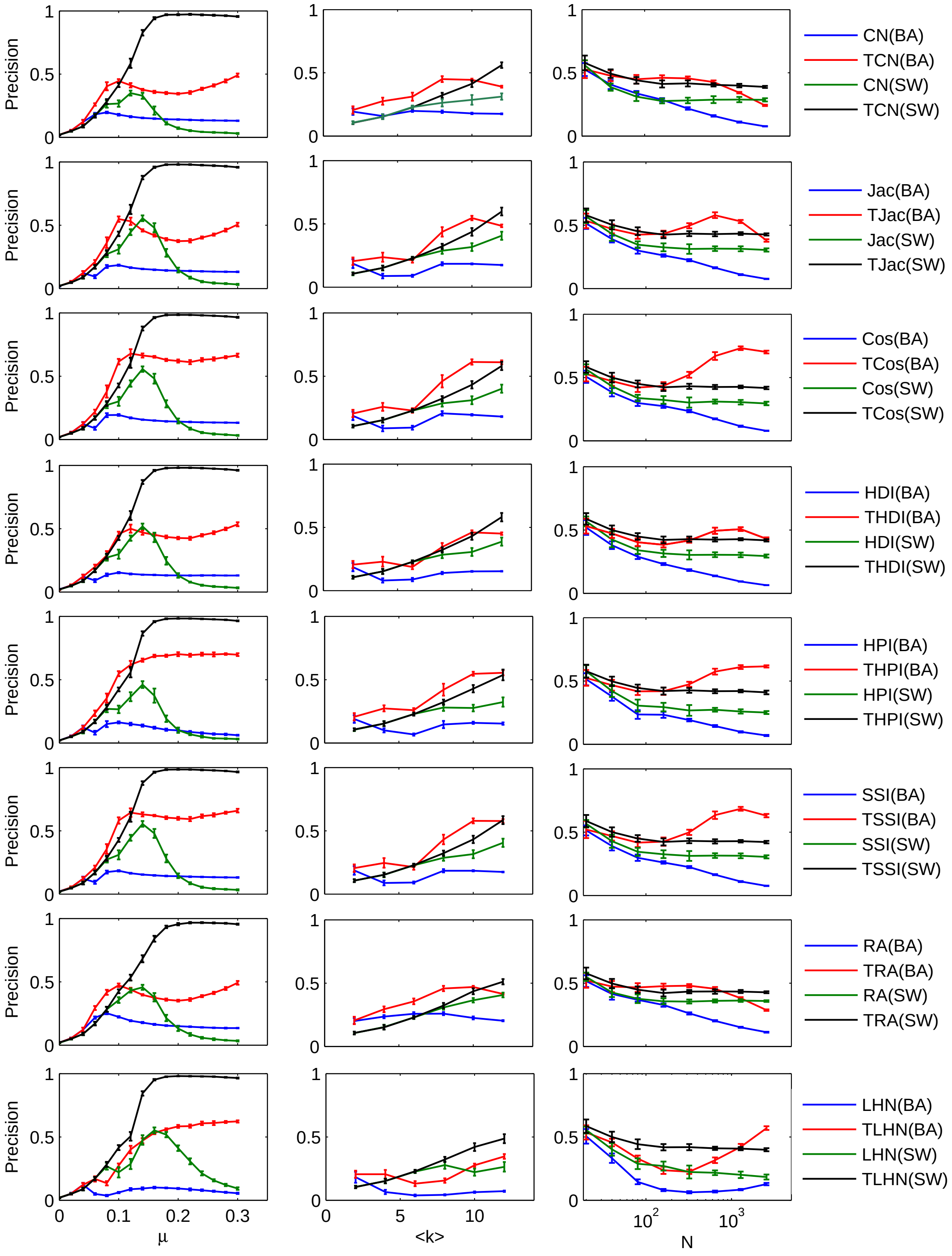

Fig. S10 The influence of different parameters (i.e. N, $\langle k \rangle$, μ) on the precision of different similarity metrics in network reconstruction. Each row of the subplots is corresponding to one similarity metric. In the first column, $\langle k \rangle = 10$, $N = 500$. In the second column, $\mu = 0.1$, $N = 500$. In the third column, $\langle k \rangle = 10$, $\mu = 0.1$.

III. SI TABLES

Table S1. Basic properties of real undirected networks and the performance of the CN, TCN, Jac and TJac methods on these networks. The parameters are set as $\mu = 2/\langle k\rangle$ and $f = 0.5$. We select a relatively large μ because the performance difference between traditional similarity metric and temporal similarity metric becomes more significant under large μ, as shown in Fig. 4. The similarity method with the best performance in each network is highlighted in bold font. The results are averaged over 50 independent realizations.

Network	AUC				Precision				Degree correlation			
	CN	TCN	Jac	TJac	CN	TCN	Jac	TJac	CN	TCN	Jac	TJac
Dolphins	0.78±0.01	**0.96±0.02**	0.83±0.01	**0.97±0.01**	0.33±0.02	0.66±0.05	0.38±0.03	**0.74±0.04**	0.67±0.04	0.77±0.07	0.69±0.05	**0.83±0.04**
Word	0.80±0.00	**0.92±0.01**	0.80±0.00	**0.93±0.01**	0.30±0.01	**0.53±0.02**	0.30±0.01	**0.55±0.02**	0.76±0.02	**0.81±0.02**	0.76±0.02	**0.87±0.02**
Jazz	0.79±0.00	**0.86±0.01**	0.79±0.00	**0.86±0.01**	0.41±0.01	**0.52±0.01**	0.42±0.00	**0.53±0.01**	**0.85±0.01**	0.82±0.01	**0.85±0.01**	0.82±0.01
E. coli	0.87±0.00	0.94±0.00	0.89±0.00	**0.97±0.00**	0.32±0.01	0.52±0.01	0.33±0.01	**0.53±0.01**	**0.83±0.02**	0.79±0.01	**0.83±0.02**	0.79±0.02
USAir	0.91±0.00	**0.93±0.00**	0.91±0.00	**0.94±0.00**	**0.52±0.00**	0.50±0.01	**0.51±0.00**	0.50±0.01	0.82±0.01	**0.84±0.01**	0.82±0.01	**0.84±0.01**
Netsci	0.86±0.01	0.98±0.00	0.97±0.00	**0.99±0.00**	0.21±0.02	0.61±0.02	0.44±0.02	**0.84±0.01**	0.50±0.03	0.63±0.03	0.64±0.02	**0.88±0.02**
Email	0.83±0.00	0.92±0.00	0.83±0.00	**0.93±0.00**	0.11±0.00	0.39±0.00	0.11±0.00	**0.40±0.00**	0.78±0.00	**0.85±0.01**	0.78±0.00	**0.85±0.01**
TAP	0.82±0.00	**0.93±0.00**	0.89±0.00	**0.99±0.00**	0.18±0.00	0.55±0.00	0.26±0.01	**0.58±0.00**	0.69±0.01	0.76±0.01	0.75±0.00	**0.78±0.00**
PPI	0.89±0.00	0.94±0.00	0.92±0.00	**0.97±0.00**	0.29±0.01	0.34±0.01	0.28±0.01	**0.35±0.01**	**0.79±0.00**	0.75±0.01	**0.79±0.01**	0.75±0.01

Table S2. Basic properties of real undirected networks(N for number of nodes, E for number of links), AUC and two network reconstruction metrics(note for Precision and Correlation) of two additional typical similarity definitions which are LHN method and RA method, and also these methods with temporal information(short for TLHN and TRA). The parameters are set as $\mu = 2/\langle k\rangle$ and $f = 0.5$. The similarity with best performance in each network is highlighted in bold font. The results are averaged over 50 independent realizations.

Networks	AUC				Precision				Degree correlation			
	LHN	TLHN	RA	TRA	LHN	TLHN	RA	TRA	LHN	TLHN	RA	TRA
Dolphins	0.76±0.04	0.94±0.01	0.82±0.03	**0.96±0.01**	0.25±0.04	0.48±0.04	0.38±0.05	**0.68±0.05**	0.07±0.06	0.44±0.16	0.57±0.06	**0.63±0.06**
Word	0.61±0.01	0.87±0.01	0.80±0.00	**0.92±0.01**	0.06±0.01	0.35±0.02	0.30±0.01	**0.53±0.03**	-0.58±0.02	-0.27±0.05	0.75±0.03	**0.82±0.03**
Jazz	0.59±0.02	0.81±0.01	0.79±0.00	**0.86±0.00**	0.10±0.02	0.38±0.01	0.42±0.01	**0.52±0.01**	-0.69±0.06	-0.05±0.09	**0.86±0.01**	0.83±0.01
E. coli	0.68±0.02	0.93±0.00	0.88±0.01	**0.95±0.00**	0.07±0.00	0.19±0.01	0.34±0.01	0.55±0.02	-0.44±0.01	-0.42±0.01	**0.82±0.02**	0.80±0.01
USAir	0.62±0.01	0.74±0.01	0.91±0.00	**0.93±0.00**	0.03±0.00	0.07±0.00	**0.52±0.00**	0.51±0.00	-0.43±0.02	-0.41±0.01	0.82±0.01	**0.84±0.01**
Netsci	0.94±0.01	**0.99±0.00**	0.94±0.01	**0.99±0.00**	0.30±0.03	0.54±0.02	0.41±0.04	**0.71±0.03**	-0.15±0.06	0.04±0.04	0.56±0.08	**0.74±0.06**
Email	0.65±0.00	**0.97±0.00**	0.83±0.00	0.92±0.00	0.03±0.00	0.18±0.01	0.12±0.00	**0.41±0.00**	-0.45±0.01	-0.46±0.01	0.78±0.00	**0.85±0.00**
TAP	0.84±0.01	**0.99±0.00**	0.87±0.01	0.96±0.00	0.14±0.01	0.30±0.01	0.25±0.04	**0.59±0.01**	-0.40±0.01	-0.47±0.01	0.73±0.04	**0.78±0.01**
PPI	0.71±0.00	**0.96±0.00**	0.90±0.00	**0.96±0.00**	0.05±0.00	0.10±0.00	0.29±0.01	**0.33±0.01**	-0.23±0.00	-0.26±0.00	**0.78±0.00**	0.72±0.01

Table S3. Basic properties of real directed networks and the performance of the CN, TCN, Jac and TJac methods on these networks. The parameters are set as $\mu = 2/\langle k\rangle$ and $f = 0.5$. We select a relatively large μ because the performance difference between traditional similarity metric and temporal similarity metric becomes more significant under large μ, as shown in Fig. 4. The similarity method with the best performance in each network is highlighted in bold font. The results are averaged over 50 independent realizations.

Networks	AUC				Precision				Degree correlation			
	CN	TCN	Jac	TJac	CN	TCN	Jac	TJac	CN	TCN	Jac	TJac
Prisoners	0.72±0.01	0.81±0.02	0.80±0.02	**0.84±0.03**	0.21±0.02	0.48±0.03	0.41±0.03	**0.58±0.02**	0.57±0.04	0.69±0.02	0.68±0.04	**0.73±0.04**
SM-FW	0.64±0.01	**0.67±0.02**	0.63±0.02	0.66±0.02	0.25±0.02	**0.29±0.02**	0.23±0.02	0.28±0.02	**0.66±0.06**	0.64±0.08	0.33±0.10	0.33±0.09
Neural	0.72±0.00	0.79±0.00	0.73±0.00	**0.81±0.00**	0.14±0.00	0.25±0.01	0.14±0.01	**0.29±0.01**	**0.68±0.00**	0.60±0.02	0.55±0.01	0.51±0.02
Metabolic	0.68±0.01	0.70±0.01	0.70±0.01	**0.72±0.01**	0.09±0.00	0.14±0.01	0.14±0.01	**0.23±0.01**	0.54±0.02	0.64±0.02	0.60±0.01	**0.71±0.02**
PB	0.84±0.00	**0.86±0.00**	0.84±0.00	**0.86±0.00**	0.15±0.00	**0.25±0.01**	0.16±0.00	**0.25±0.01**	**0.81±0.01**	0.80±0.01	0.80±0.00	0.80±0.01

Table S4. Basic properties of real directed networks(N for number of nodes, E for number of links), AUC and two network reconstruction metrics(note for Precision and Correlation) of two additional typical similarity definitions which are LHN method and RA method, and also these methods with temporal information(short for TLHN and TRA). The parameters are set as $\mu = 2/\langle k\rangle$ and $f = 0.5$. The similarity with best performance in each network is highlighted in bold font. The results are averaged over 50 independent realizations.

Networks	AUC				Precision				Degree correlation			
	LHN	TLHN	RA	TRA	LHN	TLHN	RA	TRA	LHN	TLHN	RA	TRA
Prisoners	0.73±0.02	**0.84±0.02**	0.74±0.01	0.81±0.01	0.20±0.05	**0.48±0.03**	0.24±0.02	0.46±0.02	-0.23±0.08	0.24±0.11	0.52±0.09	**0.65±0.04**
SM-FW	0.60±0.02	0.64±0.02	0.65±0.02	**0.67±0.03**	0.19±0.02	0.25±0.03	0.27±0.01	**0.31±0.01**	-0.12±0.11	0.01±0.21	**0.71±0.04**	0.70±0.04
Neural	0.66±0.01	**0.80±0.01**	0.73±0.00	**0.80±0.00**	0.07±0.01	0.24±0.01	0.17±0.01	**0.26±0.01**	-0.30±0.01	-0.24±0.02	**0.71±0.02**	0.66±0.02
Metabolic	0.68±0.01	**0.73±0.01**	0.69±0.00	0.71±0.00	0.08±0.00	0.11±0.01	0.10±0.00	**0.13±0.01**	-0.03±0.01	-0.00±0.01	0.50±0.01	**0.53±0.02**
PB	0.76±0.00	0.85±0.00	0.85±0.00	**0.86±0.00**	0.01±0.00	0.05±0.00	0.15±0.00	**0.25±0.00**	-0.22±0.00	-0.18±0.01	0.80±0.00	**0.81±0.01**

Table S5. AUC of other additional typical similarity definitions, and also these methods with temporal information. The parameters are set as $\mu = 2/\langle k\rangle$ and $f = 0.5$. The similarity with best performance in each network is highlighted in bold font. The results are averaged over 50 independent realizations.

Network	AUC											
	Cos	TCos	SSI	TSSI	HPI	THPI	HDI	THDI	PA	TPA	AS	TAS
Prisoners	0.80±0.01	0.84±0.02	0.79±0.01	0.83±0.02	0.76±0.02	0.85±0.02	0.78±0.01	0.83±0.02	0.65±0.01	0.76±0.01	0.79±0.02	**0.87±0.02**
SM-FW	0.63±0.02	0.65±0.02	0.63±0.02	0.65±0.02	0.64±0.02	0.65±0.02	0.62±0.01	0.64±0.02	0.60±0.02	0.65±0.02	0.69±0.04	**0.70±0.03**
Neural	0.74±0.00	0.83±0.00	0.73±0.00	0.82±0.00	0.74±0.00	0.84±0.01	0.72±0.00	0.80±0.00	0.71±0.00	0.76±0.00	0.78±0.00	**0.87±0.01**
Metabolic	0.71±0.01	0.73±0.01	0.70±0.01	0.72±0.01	0.73±0.01	0.75±0.01	0.70±0.01	0.72±0.01	0.64±0.01	0.69±0.00	0.75±0.01	**0.78±0.01**
PB	0.84±0.00	0.87±0.00	0.84±0.00	0.87±0.00	0.84±0.01	**0.88±0.00**	0.84±0.00	0.86±0.00	0.84±0.00	0.85±0.00	0.85±0.01	**0.89±0.00**

Table S6. Precision of other additional typical similarity definitions, and also these methods with temporal information. The parameters are set as $\mu = 2/\langle k\rangle$ and $f = 0.5$. The similarity with best performance in each network is highlighted in bold font. The results are averaged over 50 independent realizations.

Network	Precision											
	Cos	TCos	SSI	TSSI	HPI	THPI	HDI	THDI	PA	TPA	AS	TAS
Prisoners	0.40±0.04	0.57±0.02	0.40±0.04	0.57±0.02	0.11±0.03	0.56±0.03	0.38±0.04	0.57±0.02	0.16±0.02	0.32±0.03	0.16±0.02	**0.61±0.03**
SM-FW	0.24±0.02	0.28±0.02	0.23±0.02	0.27±0.02	0.22±0.02	0.29±0.03	0.22±0.01	0.27±0.02	0.25±0.02	0.27±0.01	0.25±0.02	**0.38±0.03**
Neural	0.14±0.00	0.35±0.01	0.14±0.00	0.34±0.01	0.08±0.01	0.33±0.01	0.13±0.00	0.29±0.01	0.14±0.00	0.20±0.01	0.14±0.00	**0.29±0.01**
Metabolic	0.14±0.01	**0.24±0.01**	0.14±0.01	**0.23±0.01**	0.03±0.00	0.13±0.03	0.12±0.01	0.21±0.01	0.07±0.00	0.09±0.00	0.07±0.00	0.15±0.01
PB	0.16±0.00	**0.26±0.00**	0.16±0.00	**0.26±0.00**	0.04±0.00	0.19±0.01	0.15±0.00	0.25±0.00	0.15±0.00	0.24±0.00	0.15±0.00	0.18±0.01

Table S7. Correlation of other additional typical similarity definitions, and also these methods with temporal information. The parameters are set as $\mu = 2/\langle k\rangle$ and $f = 0.5$. The similarity with best performance in each network is highlighted in bold font. The results are averaged over 50 independent realizations.

Network	Degree Correlation											
	Cos	TCos	SSI	TSSI	HPI	THPI	HDI	THDI	PA	TPA	AS	TAS
Prisoners	0.67±0.04	0.70±0.05	0.67±0.05	0.70±0.04	0.34±0.10	0.53±0.08	0.65±0.05	0.71±0.04	0.50±0.07	0.60±0.04	0.50±0.07	**0.74±0.05**
SM-FW	0.28±0.02	0.22±0.18	0.26±0.13	0.20±0.15	0.41±0.09	0.44±0.16	0.21±0.11	0.19±0.15	**0.75±0.05**	0.71±0.03	**0.75±0.06**	0.70±0.06
Neural	0.56±0.00	0.49±0.03	0.55±0.01	0.49±0.03	0.61±0.03	0.45±0.06	0.52±0.01	0.48±0.02	**0.78±0.01**	0.68±0.01	**0.78±0.01**	0.61±0.03
Metabolic	0.62±0.01	0.71±0.02	0.60±0.03	0.71±0.02	0.32±0.04	0.28±0.09	0.56±0.02	0.70±0.02	0.53±0.01	0.56±0.02	0.53±0.01	**0.79±0.03**
PB	**0.80±0.00**	0.79±0.01	**0.80±0.01**	0.79±0.01	0.74±0.01	0.55±0.04	0.80±0.01	0.80±0.01	0.80±0.00	0.80±0.01	0.80±0.00	0.77±0.01

[1] Salton, G. & McGill, M. J. Introduction to modern information retrieval (MuGraw-Hill, Auckland, 1983).
[2] Sorensen, T. A method of establishing groups of equal amplitude in plant sociology based on similarity of species and its application to analyses of the vegetation on Danish commons. *Biol. Skr.* **5**, 1-34 (1948).
[3] Ravasz, E., Somera, A. L., Mongru, D. A., Oltvai, Z. N. & Barabasi, A. L. Hierarchical organization of modularity in metabolic networks. *Science.* **297**, 1551-1555 (2002).
[4] Zhou, T., Lu, L. & Zhang, Y. C. Predicting missing links via local information. *Eur. Phys. J. B* **71**, 623?30 (2009).
[5] Zeng, A. Inferring network topology via the propagation process. *J. Stat. Mech.* **11**, 11010 (2013).
